# Analysis of the Differences in Muscle Nutrition among Individuals of Different Sexes in Redclaw Crayfish, *Cherax quadricarinatus*

**DOI:** 10.3390/metabo13020190

**Published:** 2023-01-28

**Authors:** Yuyan Sun, Xin Shan, Desheng Li, Xuxiao Liu, Zongao Han, Junjie Qin, Bin Guan, Leilei Tan, Jianbo Zheng, Min Wei, Yongyi Jia

**Affiliations:** 1 Jiangsu Key Laboratory of Marine Biological Resources and Environment/Jiangsu Key Laboratory of Marine Biotechnology, Jiangsu Ocean University, Lianyungang 222000, China; 2 Key Laboratory of Healthy Freshwater Aquaculture, Ministry of Agriculture and Rural Affairs/Key Laboratory of Freshwater Aquaculture Genetic and Breeding of Zhejiang Province, Zhejiang Institute of Freshwater Fisheries, Huzhou 313000, China; 3 Jiangsu Marine Bio-Industry Technology Collaborative Innovation Center, Jiangsu Ocean University, Lianyungang 222000, China

**Keywords:** muscle, fat, amino acids, fatty acids

## Abstract

Redclaw crayfish (*Cherax quadricarinatus*) was introduced to China many years ago. In recent years, a breeding boom for *C. quadricarinatus* has been set off in China due to a breakthrough in key technology of seedling breeding. The size and growth rate of *C. quadricarinatus* vary greatly between female and male individuals, usually the size and growth rate of male individuals are bigger than that of female individuals. There is usually a certain linkage relationship between the sex traits of crustaceans and their own nutrition. In order to explore the linkage relationship between the sex traits of *C. quadricarinatus* and its nutritional components, this study measured and analyzed the muscle nutritional components of female and male individuals. The results showed that the meat yield rate of male individuals was significantly higher than that of females (*p* < 0.05), and the crude fat content was significantly lower than that for females (*p* < 0.05). The ratios of essential amino acids to total amino acids for females and males were 39.61% and 38.49%, respectively. The ratios of essential amino acids to non-essential amino acids were 79.69% and 75.66%, respectively, which far exceed FAO/WHO standards and both belong to high-quality protein. The total amount of flavor amino acids of male individuals was significantly higher than that of female individuals (*p* < 0.05). The total amount of polyunsaturated fatty acids and the polyunsaturated fatty acid eicosapentaenoic acid of males are both significantly higher than that of females (*p* < 0.05). Studies have shown that there are certain differences in nutrition between male and female individuals. Compared with female individuals, the meat yield rate, crude protein content, and edible value of the muscles of male individuals is higher.

## 1. Introduction

*Cherax quadricarinatus*, also known as the redclaw crayfish or Australian freshwater lobster, belongs to Decapoda order, Parastacidae family, *Cherax* genus. *C. quadricarinatus* is a tropical crustacean that has become an economically important species in many countries due to its rapid growth rate, high nutritional value, and large market potential [1]. The redclaw crayfish was first introduced into Taiwan province from northern Australia in 1990 [2]. *C. quadricarinatus* has the characteristics of a large body, high growth rate, high proportion of edible parts, high protein and low fat, rich amino acid content, and it has a short breeding cycle, strong adaptability, and few diseases, so it is very suitable for artificial breeding and welcomed and loved by the farmers in China [3,4,5]. It needs a high optimum environmental temperature (27 °C); when the temperature is below 20 °C, growth arrest and high mortality occur [6,7]. At first, the lack of seedlings limited its breeding scale in China, due to the broodstocks could not survive in winter [8]. During recent years, the establishment of a large number of greenhouse nurseries has provided sufficient seed for the cultivation of red crayfish, which has promoted the development of China’s red crayfish aquaculture industry.

There are large differences between male and female of *C. quadricarinatus* in morphology and growth. The males grow faster and attain a larger size than females [9,10]. Morphologically, they exhibit male secondary characteristics, such as a red patch on the chela [11]. The males have soft projections (appendices masculinae) at the base of the fifth pair of pereiopods, while females have gonopores at the base of the third pair of pereiopods [12]. Additionally, some intersexual redclaw crayfish bearing a combination of both male and female gonopores have been found in the natural population [13,14,15,16]. Several studies have evaluated the effect of dietary protein and lipid dietary requirements on spawning rate and egg quality [17,18,19,20,21]. Moreover, the effect of dietary supplementation on fatty acid composition of *C. quadricarinatus* has been study, and the fatty acid composition in the hepatopancreas, muscle, and ovary of crayfish *C. quadricarinatus* was significantly influenced by using a combination of vitamin E, nucleotides, *Haematococcus pluvialis*, and yeast extract [22]. Moreover, the body nutritional composition of many aquatic crustacean species (e.g., shrimp and crabs) and fish had been investigated, such as Pacific krill *Euphausia pacifica*, Antarctic krill *Euphausia superba* [23], whiteleg shrimp *Penaeus vannamei*, giant freshwater prawn *Macrobrachium rosenbergii*, ridgetail white prawn *Exopalaemon carinicauda*, fleshy prawn *Fenneropenaeus chinensis*, Oriental river shrimp *Macrobrachium nipponense*, Cipango prawn *Exopalaemon annandalei*, mantis shrimp *Oratosquilla oratoria*, red swamp crayfish *Procambarus clarkii*, pink shrimp *Penaeus notialis*, and tiger shrimp *Penaeus monodon* [24,25,26]. They displayed the different nutritional composition, including routine nutrient content, fatty acid profiles, and amino acid profiles. This variation in nutritional composition is due to many factors, such as species, sex, body size, compound feed, season, and developmental stage. In particular, sex difference has a greater influence on variation in fat patterns and fatty acid patterns due to the difference in energy allocated for reproduction; females may invest 20–25% of their prebreeding weight into gonads, whereas males may invest only 3–9% [27,28]. However, relatively little is known about the research on nutritional differences between male and female redclaw crayfish.

In the present study, the comparative analysis of the conventional nutrients, amino acid components, and fatty acid components in the muscles of different sex *C. quadricarinatus* were conducted to explore the linkage relationship between nutritional components and the sex traits of *C. quadricarinatus*. The results provide a reference for the all-female or all-male *C. quadricarinatus* artificial breeding.

## 2. Materials and Methods

### 2.1. Laboratory Animal

The offspring individuals of different sexes were selected from the self-built *C. quadricarinatus* family in the laboratory, and they were similar in size, strong in vigor, and intact without injury or disease. They were cultured in a cement pond with circulating freshwater at 28 °C and fed twice a day with full-price artificial-compound feed with total protein content of 40%. The sex of sexually mature individuals were easily identified using sex secondary characteristics, such as a red patch on the chela of male individuals but no red patch on the chela of female individuals. Ten sex-mature males (body length 12.9 ± 0.7 cm; body weight 68.1 ± 7.5 g) and ten sex-mature females (body length 10.5 ± 0.5 cm, body weight 53.0 ± 3.1 g) in total were sampled and dissected for this study.

### 2.2. Determination of Meat Yield

The head and limbs of the experimental redclaw crayfish were removed, the abdomen was obtained, and the abdominal muscles were separated with a scalpel. Then, the remaining muscles on the carapace were scraped off with tweezers, and the water on the surface of the muscles was blotted with absorbent paper. The weight of the abdominal muscles was measured with an electronic balance. The calculation formula of the meat yield of shrimp is: meat yield (%) = (weight of abdominal muscle/body weight of redclaw crayfish) × 100%.

### 2.3. Routine Nutrient Content Determination

The *C. quadricarinatus* were divided into two groups (male and female groups), and the abdominal muscle of the redclaw crayfish was taken as the sample to determine the content of water, crude ash, crude protein, and crude fat. The moisture was determined by a direct drying method [29], and the crude ash content was determined by 550 °C burning for 24 h in a muffle furnace [30]. The crude protein was determined by using an automatic Kjeldahl nitrogen analyzer (KjeltecTM-8400, FOSS, Höganäs, Sweden) according to the experimental instructions, and the crude fat was determined by a Soxhlet extraction apparatus (SoxtecTM-8000 Extraction Unit, FOSS, Suzhou, China) according to the method for using the instrument.

### 2.4. Amino Acid Composition Determination and Analysis

Amino acids were determined by the 6 N hydrochloric acid hydrolysis method [31], and a Hitachi Amino Acid Analyzer (L-8900, Hitachi, Kyoto, Japan) was used to analyze the amino acid composition of the abdominal muscle from *C. quadricarinatus*.

### 2.5. Fatty Acid Composition Analysis

The total fat in the samples was extracted according to published methods [32], and the fatty acid composition was determined by using a Shimadzu instrument (GC-MS 2010 SE, Kyoto, Japan). The map was queried against the National Institute of Standards and Technology (NIST) database (https://www.nist.gov/, accessed on 4 February 2022) for qualitative search, and the area normalization method was used for percentage quantification.

### 2.6. Data Processing and Analysis

SPSS 23.0 software was used to conduct independent samples T-test on the data, and the normality and homoscedasticity of the data were also tested during the statistical analysis; *p* < 0.05 indicated significant difference, and the data are presented as mean ± standard error (mean ± SE).

## 3. Results

### 3.1. Analysis of Abdominal Muscle Meat Yield and Conventional Nutritional Components of C. quadricarinatus with Different Sexes

The measurement results of the *C. quadricarinatus* meat yield showed that the meat yield of male individuals was about 19.12% (Table 1), which was significantly higher than that of the female individuals (15.81%) (*p* < 0.05).

The results of conventional nutrient composition analysis of male and female individuals showed (Table 1) that the muscle moisture and crude fat content of male individuals were significantly lower than those of female individuals (*p* < 0.05), and the crude ash content was significantly higher than that of female individuals (*p* < 0.05).

### 3.2. Composition and Content of Amino Acids in Abdominal Muscles of C. quadricarinatus with Different Sexes

The analytical results of amino acid components of *C. quadricarinatus* with different sexes showed that a total of 17 amino acids were detected in the muscle of *C. quadricarinatus* (Table 2), including 7 human essential amino acids (EAA: Thr, Val, Met, Phe, Ile, Leu, Lys) (tryptophan was not detected due to acid hydrolysis damage), 2 semi-essential amino acids (SEAA: His, Arg) and 8 non-essential amino acids (NEAA: Asp, Ser, Glu, Gly, Ala, Cys, Pro, Tyr).

The content of glutamic acid (Glu) in the abdominal muscle of *C. quadricarinatus* was the highest, and it reached the highest value (4.03 mg/g) in male individuals, which was significantly higher than that of female individuals (*p* < 0.05). The contents of valine (Val), lysine (Lys), aspartic acid (Asp), and tyrosine (Tyr) were significantly different between different sex individuals, and the contents of Asp and Lys in male individuals were significantly higher than those in female individuals (*p* < 0.05), while tyrosine was significantly lower than that of female individuals (*p* < 0.05). In addition, there was no significant difference in the total content of amino acids (ƩTAA), the total content of essential amino acids (ƩEAA), the total content of non-essential amino acids (ƩNEAA) and the total content of umami amino acids (ƩDAA) between male and female individuals (*p* > 0.05).

### 3.3. Composition and Content of Fatty Acids in Abdominal Muscles of C. quadricarinatus with Different Sexes

The determination of fatty acid composition (using 37 kinds of fatty acid methyl ester standards) showed that 12 kinds of fatty acids were detected in the *C. quadricarinatus* muscle (Table 3), and the remaining 25 kinds of fatty acids were not detected due to too low content. Among these 12 fatty acids, the ratio of saturated fatty acids (C12:0, C14:0, C15:0, C16:0, C17:0, and C18:0) to unsaturated fatty acids (C16:1, C18:1n-9t, C18:1n-9c, C18:2n-6t, C20:5n-3, and C22:6n-3) is 1:1, and the ratio of monounsaturated fatty acids (C16:1, C18:1n-9t, and C18:1n-9c) and polyunsaturated fatty acids (C18:2n-6t, C20:5n-3, and C22:6n-3) is also 1:1. The specific composition of fatty acids was analyzed, and the results showed that the total content of saturated fatty acids (ΣSFAs) in individuals with different sexes was not significantly different. The content of C16:0 in males was higher than that in females (*p* > 0.05), and C12:0, C14:0, C17:0, and C18:0 were significantly lower than those in females (*p* < 0.05).

The total content of monounsaturated fatty acids (ΣMUFAs) of male *C. quadricarinatus* were significantly lower than that of females (*p* < 0.05), and the monounsaturated fatty acids were mainly C18:1n-9c, accounting for 13.89~14.52% of the total fatty acids in abdominal muscle of *C. quadricarinatus*. The contents of the three monounsaturated fatty acids (C16:1, C18:1n-9t, and C18:1n-9c) of female individuals were significantly higher than those of male individuals (*p* < 0.05), while the total content of polyunsaturated fatty acids (ΣPUFAs) of male individuals was significantly higher than female individuals (*p* < 0.05). Among these polyunsaturated fatty acids, the content of DHA of male individuals was slightly lower than females, while the contents of C18:2n6t and EPA in males were significantly higher than those of females (*p* < 0.05).

## 4. Discussion

### 4.1. Analysis of Meat Yield and Conventional Nutrition Difference

As an important indicator to measure the quality of aquatic animals, meat yield is affected by various factors such as individual features, growth environment, and nutrient intake. In this study, the meat yield of *C. quadricarinatus* is about 10%, which is lower than the result previously reported [33]. The reason it is about 10% may be related to the calculation; the meat yield of *C. quadricarinatus* includes the muscles of the first step foot and abdomen.

The crude fat content in the muscles of the female and male individuals are 1.73% and 1.53%, respectively, and the crude fat content is significantly higher in female individuals than that in male individuals (*p* < 0.05). The crude protein content reach more than 80%, and the crude protein content of male individuals is higher (84.16%) than that of female individuals (*p* > 0.05), which is significantly higher than that of marine aquaculture Japanese eel *Anguilla japonica* (55.02%), freshwater aquaculture Japanese eel *Anguilla japonica* (57.84%), Pacific krill *E. pacifica* (65.80%), and Antarctic krill *E. superba* (62.80%) [34,35]. It has been reported that the nutritional composition of animals is closely related to food sources, composition, and their own growth stages [36,37]. All of the *C. quadricarinatus* used in this study were sampled from the same breeding base and at the same growth stage, and were fed with the same compound feed. Therefore, it can be speculated that the differences in the conventional nutritional components between different sex individuals is not related to their growth stages and intakes, mainly due to the different genetic basis caused by gender differences, indicating that there is a certain degree of correlation between gender and the content of nutrients in the muscle of *C. quadricarinatus*. Females typically carry more fat than males to produce large and energy-rich macrogametes and facilitate successful offspring, while males may need more robust bones to give them an advantage in mate searches and battles [27,28,38].

### 4.2. Differential Analysis of Amino Acid Composition

The protein quality of most aquatic animals is affected by the type and content of amino acids, and the type and content of amino acids play an important role in the growth and development of aquatic animals and their quality evaluation [39]. Additionally, according to the high-quality food protein standards formulated by the Food and Agriculture Organization of the United Nations (FAO) and the World Health Organization (WHO) [40], high-quality protein not only contains a complete range of essential amino acids, but also has a suitable ratio between essential amino acids [41]. The high-quality protein standard of FAO/WHO is that the ratio of ΣEAA/ΣTAA is more than 40%, and the ratio of ΣEAA/ΣNEAA is more than 60% [42]. The average ratios of ΣEAA/ΣTAA in male and female *C. quadricarinatus* were 39.61% and 38.49%, very close to 40%, while the ratio of ΣEAA/ΣNEAA was 79.69% and 75.66%, which is higher than the high-quality protein standards set by FAO and WHO. The results show that the *C. quadricarinatus* shrimp meat is generally a high-quality protein source, and the amino acid balance effect of female individuals is better than that of male individuals in this study.

The study also found that the composition and content of the umami amino acids (DAA: Asp, Glu, Gly, Ala, Tyr, Phe) in protein play a major role in the taste of aquatic animal meat. The amino acids that can bring umami characteristics are Asp and Glu [43], and Gly and Ala are characteristic amino acids of sweet taste [44]. Among them, the proportion of male umami amino acids was as high as 47.33%, higher than most aquatic animals [45].

According to literature reports, glutamate can reduce the ammonia content in the blood, and it is the main component in the drug for the treatment of hepatic coma, and has great medical value [46]. In this study, the content of glutamate in *C. quadricarinatus* individuals was higher, and the glutamate in the muscle of male individuals was significantly higher than that of female individuals (*p* < 0.05). Therefore, the male *C. quadricarinatus* has better meat flavor and higher medicinal value, and can be used as an ingredient choice for nutritional meals for patients with liver disease. In addition, male *C. quadricarinatus* has a large physique, high meat yield, and is more popular for the consumption market.

The EAAs and NEAAs are needed in diets to improve the growth, development, molt rate, survival, and reproduction of crustaceans (shrimp and crabs) [23]. Indeed, the AAs, particularly aspartate and glutamate, are important metabolic fuels in the ovaries of blue crabs (*Callinectes sapidus*) [23]. Shrimp (*Litopenaeus setiferus*) fed with a 35% protein diet had a lower sperm quality than shrimp fed with a 45% protein diet, suggesting that dietary AAs are important for its male sperm development [47]. The results indicate that the redclaw crayfish *C. quadricarinatus* might have particular biochemical pathways for these differential AAs, dependent on sex, which ultimately could reflect sex-specific AA needs.

### 4.3. Differential Analysis of Fatty Acid Composition

Experimental studies have found that eating a large amount of saturated fatty acids can lead to an increase in the activity of 3-hydroxy-3-methylglutaryl coenzyme A (HMG-CoA) reductase in the liver, which increases cholesterol synthesis [48,49]. Therefore, excessive intake of saturated fatty acids is the main reason for the increase in blood cholesterol, triacylglycerol, and low-density lipoprotein cholesterol, and secondary to the stenosis of the arterial lumen, the formation of atherosclerosis, and the increased risk of coronary heart disease [50,51,52]. The study found that there was no significant difference in the content of total saturated fatty acids in the *C. quadricarinatus* of the two sexes, and the content in females was the highest (56.72%), but it was also far lower than that of the South African *Penaeus monodon* (62.84%) and the *Fenneropenaeus chinensis* (64.36%) [53]. Therefore, considering the saturated fatty acid content, *C. quadricarinatus* has more practical value and health care effect than South African *P. monodon* and *F. chinensis*.

Among the polyunsaturated fatty acids, EPA and DHA are generally regarded as important indicators to measure the value of fat [54,55]. EPA is an important unsaturated fatty acid that cannot be synthesized by the body itself but is indispensable, so it must be directly supplemented from food; DHA, commonly known as brain gold, is an important fatty acid associated with promoting brain development and intellectual development. In the present study, the EPA (9.8%) in the muscle of the male *C. quadricarinatus* was significantly higher than that of the female *C. quadricarinatus* (*p* < 0.05), while the content of DHA (3.27%) was slightly lower than that of female (3.56%), but there was no significant difference (*p* > 0.05). Therefore, in terms of the content of EPA and DHA, male redclaw crayfish have more edible and aquaculture value. The hypothesis that PUFAs, in particular EPA, play vital roles in the sexual maturation of the Baltic herring *Clupea harengus membras* has been confirmed [56]. Fatty acid profiles varied between sexes in the *C. quadricarinatus*, with females containing a greater proportion of SFA and MUFA, while males have more polyunsaturated fatty acids in quantity, especially when expressed on a edible part (muscle rather than adipose tissue) basis. Similar results have been reported for cultured brook trout *Salvelinus fontinalis*, Black Sea trout *Salmo tutta labrax* [57], Nile tilapia *Oreochromis niloticus* [58], sea lamprey *Petromyzon marinus* [59], and African clawed frog *Xenopus laevis* [60], which indicate that different sexes may possess different mechanisms for the metabolism of certain fatty acids.

## 5. Conclusions

This study explored the relationship between sex and muscle nutrition. The results show that there is a certain relationship between sex and muscle nutrition. Female individuals carry more fat than male individuals to produce energy-rich macrogametes and facilitate successful offspring, while male individuals have larger body size and higher meat yield than female individuals. The male redclaw crayfish might have particular biochemical pathways, and reflect sex-specific amino acids (especially in umami amino acids, aspartic acid, and glutamic acid) and fatty acids (especially in polyunsaturated fatty acid and eicosapentaenoic acid) demand.

## Figures and Tables

**Table 1:** The meat rate and content of conventional nutrients in the muscles of *C. quadricarinatus* with different sexes (dry weight; N = 10).

Items	Males	Females
Meat rate (%)	19.12 ± 0.61^a^	15.81 ± 0.36^b^
Moisture (%)	78.15 ± 0.26^b^	79.37 ± 0.30^a^
Ash (%)	6.34 ± 0.06^a^	5.59 ± 0.05^b^
Crude fat (%)	1.73 ± 0.05^a^	1.57 ± 0.04^b^
Crude protein (%)	84.16 ± 0.84	81.56 ± 2.18

Notes: values (mean ± SE) in the same row with different lowercase superscripts represent significant difference (*p* < 0.05).

**Table 2:** Analysis of amino acid composition in the muscles of *C. quadricarinatus* with different sexes (dry matter; N = 10).

Amino Acid	Males (%)	Females (%)
Thr *	0.62 ± 0.06	0.48 ± 0.03
Val *	0.63 ± 0.04^a^	0.47 ± 0.03^b^
Met *	0.42 ± 0.02	0.35 ± 0.06
Ile *	0.71 ± 0.05	0.74 ± 0.07
Leu *	1.79 ± 0.17	1.73 ± 0.30
Lys *	1.71 ± 0.38^a^	1.32 ± 0.13^b^
Phe *^&^	0.57 ± 0.05	0.61 ± 0.03
His ^△^	0.4 ± 0.03	0.34 ± 0.05
Arg ^△^	1.24 ± 0.21	1.2 ± 0.05
Asp ^#&^	1.64 ± 0.23^a^	1.35 ± 0.13^b^
Ser ^#^	0.64 ± 0.04	0.60 ± 0.03
Glu ^#&^	4.03 ± 0.57^a^	2.60 ± 0.27^b^
Gly ^#&^	0.52 ± 0.05	0.51 ± 0.08
Ala ^#&^	0.77 ± 0.05	0.86 ± 0.07
Cys ^#^	0.37 ± 0.02	0.32 ± 0.01
Tyr ^#&^	0.53 ± 0.04^b^	0.73 ± 0.05^a^
Pro ^#^	0.33 ± 0.02	0.19 ± 0.07
ΣEAA	6.46 ± 0.46	5.71 ± 0.13
ΣNEAA	8.87 ± 0.70	7.18 ± 0.33
ΣTAA	16.91 ± 1.02	14.42 ± 0.50
ΣDAA	8.06 ± 0.72	6.67 ± 0.40
ΣEAA/ΣNEAA	75.66 ± 6.59	79.69 ± 2.71
ΣEAA/ΣTAA	38.49 ± 2.08	39.61 ± 0.84
ΣDAA/ΣTAA	47.33 ± 2.44	46.15 ± 1.20

Notes: * represents essential amino acids; ^△^ represents semi-essential amino acids; ^#^ represents non-essential amino acids; ^&^ represents umami amino acids; ƩTAA shows total content of amino acids (TAA); ƩEAA shows total content of essential amino acids (EAA); ƩNEAA shows total content of non-essential amino acids (NEAA); ΣDAA shows total content of umami amino acids (DAA). Values (mean ± SE) in the same row with different lowercase superscripts represent significant difference (*p* < 0.05).

**Table 3:** Analysis of fatty acids composition in the muscles of *C. quadricarinatus* with different sexes (dry matter; N = 10).

Fatty Acid	Males (%)	Females (%)
C12:0	0.4 ± 0.05^b^	0.73 ± 0.21^a^
C14:0	1.24 ± 0.1^b^	1.43 ± 0.21^a^
C15:0	0.74 ± 0.06	0.79 ± 0.02
C16:0	34.82 ± 0.09	33.64 ± 0.42
C17:0	1.44 ± 0.01^b^	1.58 ± 0.05^a^
C18:0	18.06 ± 0.12^b^	18.55 ± 0.12^a^
ΣSFAs	56.69 ± 0.28	56.72 ± 0.17
C16:1	0.75 ± 0.01^b^	0.91 ± 0.06^a^
C18:1n-9t	1.46 ± 0.05^b^	1.71 ± 0.09^a^
C18:1n-9c	13.89 ± 0.24^b^	14.52 ± 0.16^a^
ΣMUFAs	16.11 ± 0.28^b^	17.14 ± 0.3^a^
C18:2n-6t	14.12 ± 0.2	13.43 ± 0.17
EPA C20:5n-3	9.8 ± 0.45^a^	9.15 ± 0.32^b^
DHA C22:6n-3	3.27 ± 0.6	3.56 ± 0.19
ΣPUFAs	27.2 ± 0.24^a^	26.13 ± 0.39^b^
ΣMUFAs + ΣPUFAs	43.31 ± 0.28	43.28 ± 0.17

Notes: Fatty acids with small content are not listed in the table. Values (mean ± SE) in the same row with different lowercase superscripts represent significant difference (*p* < 0.05).

## Data Availability

All data generated or analyzed during this study are included in this published article.
